# Efficacy of a Y-modified Transconjunctival Approach for Open Reduction and Internal Fixation of Zygomaticomaxillary Complex Fractures: Functional Recovery and Cosmetic Outcomes

**DOI:** 10.7759/cureus.96679

**Published:** 2025-11-12

**Authors:** Sujeeth K Shetty, Rishabh Kasrija

**Affiliations:** 1 Department of Oral and Maxillofacial Surgery, Jagadguru Sri Shivarathreeshwara (JSS) Dental College and Hospital, Mysuru, IND

**Keywords:** cosmetic incision, cosmetic procedures, modification, ­wound healing, zygomatico-orbital complex

## Abstract

Introduction: Fractures of the zygomaticomaxillary complex (ZMC), a crucial component of the midface, frequently lead to functional impairments, such as diplopia, enophthalmos, and restricted ocular motility. Although surgical intervention is often required, conventional techniques can be limited by suboptimal access and potential aesthetic drawbacks. This study prospectively assessed the efficacy, functional recovery, and cosmetic results of the Y-modified transconjunctival technique for managing ZMC fractures with concomitant orbital floor injury.

Materials and methods: A prospective cohort study was conducted at a single oral and maxillofacial surgery department. Fifteen eligible patients (aged 18-65 years) presenting with displaced ZMC fractures and orbital floor defects were treated with open reduction and internal fixation using the Y-modified transconjunctival approach. Outcome measures included pain (Visual Analog Scale (VAS) score), enophthalmos (Hertel exophthalmometer), scar quality (Patient and Observer Scar Assessment Scale (POSAS) 3.0), and paresthesia. Fracture reduction and implant position were verified using high-resolution computed tomography. Patients were systematically evaluated postoperatively on days 1, 10, 28, and 42, and the data were subjected to statistical analysis.

Results: The results demonstrated a statistically significant reduction in pain levels over time, with mean VAS scores declining from 5.82 on postoperative day 1 to 1.00 by day 42 (p < 0.01). Residual enophthalmos was observed in a single case (6.67%). Objective scar evaluation using POSAS 3.0 indicated marked improvements in vascularity, pigmentation, and pliability between the 10- and 28-day assessments (p < 0.01). Furthermore, patient-reported feedback corroborated these findings, indicating a diminished perception of discomfort and a positive view of scar healing.

Conclusions: The Y-modified transconjunctival approach proved to be an effective strategy for ZMC fractures with orbital floor involvement, providing ample surgical exposure for stable fixation while yielding excellent functional and aesthetic outcomes. However, the promising results should be interpreted in the context of the study's limitations, including a modest sample size and single-institution design. Future research involving larger multicenter cohorts with long-term follow-up is necessary to confirm these preliminary findings and establish broader clinical applicability.

## Introduction

The zygomaticomaxillary complex (ZMC) is a critical anatomical structure of the midface that contributes to cheek prominence, orbital integrity, and overall facial aesthetics and function [1]. ZMC fractures are among the most common maxillofacial injuries and typically result from high-impact trauma, such as motor vehicle accidents or assaults, owing to the prominent anatomical projection [2,3]. These fractures are challenging to manage due to their involvement of multiple anatomical planes, including the zygomatic arch, orbital floor, and maxillary sinus. Inaccurate treatment can lead to complications such as diplopia, enophthalmos, and facial asymmetry [4,5]. Effective surgical intervention requires precise reduction and stable fixation to restore form and function, given the complex relationship between the zygoma and the surrounding structures [6].

Treatment strategies for ZMC fractures range from conservative management for minimally displaced fractures to surgical intervention for complex cases [7]. Open reduction and internal fixation (ORIF) using miniplates remains the gold standard, with approaches such as coronal, buccal sulcus, or transconjunctival being commonly employed [8]. The transconjunctival approach has gained favor due to its minimally invasive nature, offering direct access to the orbital floor and infraorbital rim while minimizing external scarring. However, its limited exposure can restrict visualization and manipulation of the zygomatic complex, particularly in cases with significant orbital floor discontinuity, where precise restoration of the orbital volume is critical [9,10].

To overcome these limitations, the Y-modification of the transconjunctival approach incorporates a lateral extension, creating a Y-shaped incision that enhances surgical exposure. This technique enhances access to the zygomatic body and orbital floor while preserving the aesthetic benefits of the transconjunctival route [7,11]. Y modification facilitates accurate reduction and fixation, which are essential for preventing postoperative complications and restoring orbital volume. Compared with subciliary approaches, it reduces the risk of ectropion. It maintains minimal visible scarring, although it may increase operative time and carry a risk of conjunctival injury, as well as a steeper learning curve [11,12].

Although prior studies have described the advantages of Y modification, few have systematically evaluated its outcomes in the context of complex ZMC fractures with significant orbital floor discontinuity, a subgroup in which adequate exposure is particularly critical [7,13]. Earlier research often focused on general ZMC fracture management or traditional transconjunctival approaches, with limited emphasis on this challenging subset or on comprehensive outcome assessment [9]. This study introduced a novel focus by specifically investigating the efficacy of Y modification in managing ZMC fractures with substantial involvement of the orbital floor. Unlike previous studies, this study employed a detailed evaluation of both functional outcomes (e.g., restoration of orbital volume and resolution of diplopia) and aesthetic outcomes (e.g., facial symmetry and patient-reported satisfaction) using standardized metrics. By addressing this understudied subgroup and providing a holistic outcome analysis, this study aimed to fill a critical gap in the literature, offering evidence to guide surgical decision-making and optimize patient care in maxillofacial trauma.

## Materials and methods

Study design

This prospective cohort study was conducted at the Department of Oral and Maxillofacial Surgery, Jagadguru Sri Shivarathreeshwara (JSS) Dental College, Mysore, between June 2023 and December 2024, to evaluate the clinical, functional, and aesthetic outcomes of the Y-modified transconjunctival approach for managing ZMC fractures with orbital floor discontinuity. The study protocol was approved by the Institutional Ethical Committee of JSS Dental College (approval number: 19/2023), and written informed consent was obtained from all enrolled participants prior to their inclusion. This study adhered to the principles of the Declaration of Helsinki.

Patient selection

Patients aged 18-65 years presenting with unilateral or bilateral ZMC fractures and orbital floor discontinuity confirmed by computed tomography (CT) scans were included. The inclusion criteria were displaced ZMC fractures requiring ORIF and orbital floor defects necessitating reconstruction. Exclusion criteria included previous orbital or zygomatic surgery, severe comorbidities (e.g., cardiac and respiratory), ocular pathology, infected fracture of the orbito-ZMC region, comminuted ZMC fractures, or refusal to consent.

A sample size of 15 patients was calculated to detect a clinically meaningful difference in the primary outcome of enophthalmos correction (measured by Hertel exophthalmometry) with 80% power and a two-sided α of 0.05. A mean difference of 2 mm in enophthalmos between preoperative and postoperative measurements was considered clinically significant, with an estimated standard deviation of 2.5 mm, based on institutional pilot data from five patients treated with the Y-modified transconjunctival approach at JSS Dental College and clinical consensus that a 2 mm change is functionally and aesthetically relevant in ZMC fracture repair [14]. Using the formula for the paired t-test, the calculated sample size was n = 12.3. Accounting for a 20% dropout rate, the final sample size was rounded off to 15 patients.

Preoperative assessment and evaluation

Patients presenting with suspected ZMC fractures underwent comprehensive preoperative evaluation. Radiological examination was conducted for all selected subjects using conventional radiographs, including the submentovertex view and paranasal sinus view, and/or advanced imaging modalities, such as CT of facial bones, to confirm the diagnosis and assess the extent of ZMC fractures with orbital floor discontinuity. Additionally, routine hematological investigations and serology were performed for all subjects to evaluate their overall health status. The subjects were also screened to ensure fitness for surgery under general anesthesia, adhering to standard preoperative protocols.

A detailed medical history, including the mechanisms of injury, associated trauma, and comorbidities, was collected. Clinical examination assessed facial symmetry, ocular motility, diplopia, enophthalmos (using a calibrated Hertel exophthalmometer; Oculus Inc., Wetzlar, Germany), and sensory deficits along the infraorbital nerve distribution. An ophthalmologist evaluated visual acuity and globe integrity to rule out ocular pathologies.

Radiographic assessment was performed using a high-resolution CT scanner (Somatom Definition Edge, Siemens Healthineers, Erlangen, Germany) to confirm ZMC fractures with orbital floor discontinuity and to classify fracture displacement. Scans were acquired in the axial, coronal, and sagittal planes with a slice thickness of 1 mm, a field of view of 200 mm, a bone reconstruction algorithm (B70s kernel), a tube voltage of 120 kVp, a tube current of 200 mAs, and a pitch of 0.8.

All clinical assessment tools were calibrated before use to ensure measurement accuracy. The Hertel exophthalmometer (Oculus Inc., Wetzlar, Germany) was calibrated according to the manufacturer’s guidelines before each session to verify the alignment and precision in measuring enophthalmos. For clinical assessments, including pain (Visual Analog Scale (VAS) score) [15], scar evaluation using the Patient and Observer Scar Assessment Scale (POSAS) 3.0 [16], and sensory testing along the infraorbital nerve distribution, reliability was evaluated to ensure consistency. Interobserver reliability was assessed by having two independent examiners perform measurements on a subset of 10 patients during the same visit. The intraclass correlation coefficient (ICC) was calculated, with values > 0.75 considered acceptable. For the POSAS 3.0, both patient and observer components (vascularity, pigmentation, thickness, relief, pliability, pain, and itching) were evaluated for their level of agreement. Sensory testing for paresthesia was standardized using a two-point discrimination test and light touch assessment with a cotton wisp.

Surgical procedure

**Figure 1:**
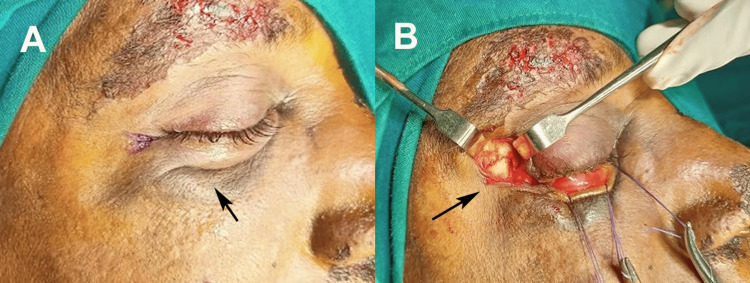
Intraoperative views of ZMC fracture management. (A) Preoperative marking of the lateral eyebrow incision (arrow) used to access the frontozygomatic suture region. (B) Surgical exposure of the zygomaticomaxillary fracture site through the marked incision (arrow) showing underlying bone and fracture line visualization. ZMC: zygomaticomaxillary complex Written informed consent was obtained from the patient for the publication of the original image.

Following nasoendotracheal or oral intubation, the surgical field was prepared aseptically using a 5% povidone-iodine solution (Betadine, Purdue Pharma, Stamford, CT, USA) and draped according to standard protocol. A forced duction test was performed to assess extraocular muscle entrapment and restrictions in globe mobility. A corneal shield (EyeGard, Bausch & Lomb, Rochester, NY, USA) with a lubricating solution was placed to protect the eye during surgery. A Y-shaped incision was made in the lateral canthal area following the natural skin crease. Local infiltration was performed with 2% lignocaine containing 1:200,000 epinephrine (Xylocaine; AstraZeneca, Cambridge, UK) to achieve vasoconstriction and reduce intraoperative bleeding in the conjunctival and lateral canthal regions (Figure 1).

The lower eyelid was everted and stabilized using three traction sutures (5-0 Prolene, Ethicon Inc., Somerville, NJ, USA) placed through the tarsus medially, centrally, and laterally. The skin incision followed a marked Y-pattern, with the superficial limb of the lateral canthal tendon incised to allow separation of the tarsal plates. An incision was made through the conjunctival mucosa, and a subconjunctival tunnel was created toward the medial end using a hemostat. Conjunctival sectioning was performed 5 mm below the lower tarsal plate using surgical scissors. The orbicularis oculi muscle was identified and incised in line with the infraorbital rim. Subsequent sectioning of the orbital septum on the maxillary facial surface was followed by subperiosteal dissection to expose the infraorbital rim and the floor.

**Figure 2:**
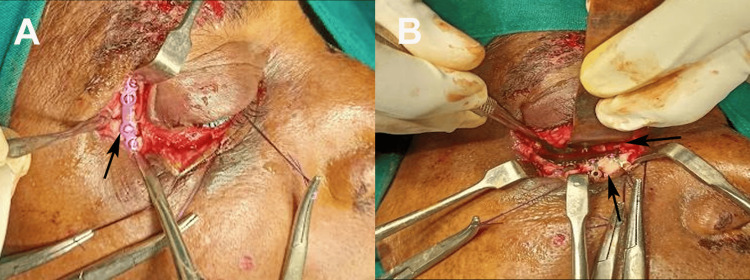
Intraoperative fixation of ZMC fracture. (A) Placement of a titanium miniplate (arrow) at the frontozygomatic suture region to stabilize the zygomatic segment. (B) Fixation of the zygomaticomaxillary buttress with titanium plates and screws (arrows). ZMC: zygomaticomaxillary complex Written informed consent was obtained from the patient for the publication of the original image.

To facilitate Y-modification, lateral canthotomy was performed by dividing the superficial limb of the lateral canthal ligament, which allowed expansion of the incision into a box-like configuration. This enhanced the exposure of the frontozygomatic suture, the lateral orbital wall, the infraorbital rim, and the orbital floor. A maxillary vestibular incision was made from the canine to the first molar to access and reduce the zygomatic buttress fracture. The fracture was anatomically reduced along both vertical and horizontal planes at the frontozygomatic suture, infraorbital rim, and zygomatic buttress. The entrapped orbital fat or muscles are released and repositioned within the orbit. Fixation was achieved using 1.5 mm titanium miniplates and screws (MatrixNEURO, DePuy Synthes, Raynham, MA, USA) and a contoured titanium mesh (Orbital Floor Mesh, DePuy Synthes, Raynham, MA, USA) to support orbital contents. A final forced duction test was performed to ensure decompression (Figure 2).

**Figure 3:**
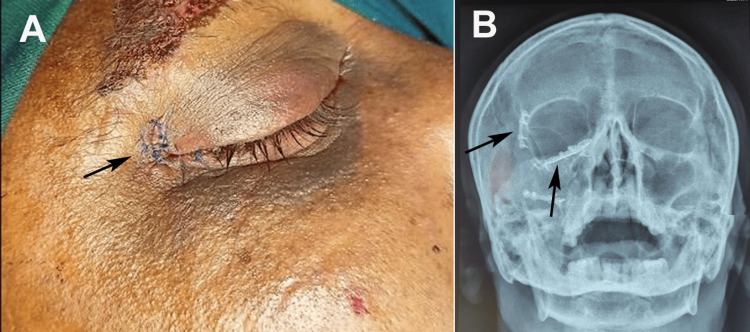
Postoperative evaluation of ZMC fracture. (A) Clinical photograph showing the surgical incision with sutures at the lateral canthal region (arrow), minimal swelling, and satisfactory skin closure. (B) Postoperative radiograph (Waters view) demonstrating reduction of the ZMC fracture with fixation hardware visible at the zygomatic buttress and infraorbital rim (arrows). ZMC: zygomaticomaxillary complex Written informed consent was obtained from the patient for the publication of the original image.

In cases involving concurrent mandibular or maxillary fractures, appropriate reduction and fixation were performed using 2 mm miniplates and screws (MatrixMANDIBLE, DePuy Synthes, Raynham, MA, USA) under maxillomandibular fixation (MMF). Hemostasis was achieved, and the surgical field was irrigated with antiseptic (Betadine) and saline solutions. Closure was performed in the following layers: lateral canthal ligament and orbicularis oculi with 4-0 polyglactin 910 (Vicryl, Ethicon Inc., Somerville, NJ, USA), conjunctiva with 5-0 polyglactin 910 (Vicryl), intraoral mucosa with 4-0 polyglactin 910 (Vicryl), and skin with 5-0 polypropylene (Prolene, Ethicon Inc., Somerville, NJ, USA) in a Y configuration. Traction sutures were removed, the sclera was irrigated with saline, and neomycin ophthalmic ointment (Neosporin; GlaxoSmithKline, Brentford, UK) was applied with eye padding (Figure 3).

Postoperative care

All patients received intravenous antibiotics, including amoxicillin-clavulanic acid (Augmentin, 1.2 g twice daily, GlaxoSmithKline, Brentford, UK) and metronidazole (Flagyl, 500 mg three times daily, Pfizer, New York, NY, USA), for five days to prevent postoperative infection, as per institutional protocols for maxillofacial surgery. Pain was managed with paracetamol (crocin, 1 g twice daily; GlaxoSmithKline, Brentford, UK) for five days. Patients were advised to avoid strenuous activities and maintain a soft diet for two weeks.

Outcome assessment

Patients were followed up on postoperatively on the 1st, 10th, 28th (four weeks), and 42nd (six weeks) to undergo clinical and radiographic evaluations. Clinical evaluation was performed at each follow-up visit to assess multiple outcomes. Pain was measured using an open-access VAS (rated 0-10) [15]. Scar assessment was conducted using an open-access POSAS 3.0 [16] on the 10th and 28th postoperative days (PODs) to evaluate multiple parameters under standardized conditions (Table 1).

**Table 1:** Patient and Observer Scar Assessment Scale (POSAS) 3.0. The Patient and Observer Scar Assessment Scale (POSAS) 3.0 is available for non-commercial use under an open-access agreement [16].

Scar parameter	Assessment method	Scoring scale
Vascularity	Visual inspection under uniform lighting; blanching technique with finger or glass slide	1 = normal (same as surrounding skin), 2 = pink, 3 = red, 4 = purple, 5 = hyper-vascular (excessively red)
Pigmentation	Visual evaluation under standardized lighting	1 = normal (same pigmentation as surrounding skin), 2 = slightly hypo/hyperpigmented, 3 = moderately hypo/hyperpigmented, 4 = severely hypo/hyperpigmented, 5 = extremely hypo/hyperpigmented
Thickness	Visual inspection and palpation	1 = normal (same as surrounding skin), 2 = slightly thicker or thinner, 3 = moderately thicker or thinner, 4 = severely thicker or thinner, 5 = extremely thicker or thinner (hypertrophic/keloid or atrophic)
Relief	Visual inspection and palpation for ridges, depressions, or nodules	1 = normal (even with surrounding skin), 2 = slightly irregular, 3 = moderately irregular, 4 = severely irregular, 5 = extremely irregular (deep pits or raised nodules)
Pliability	Gentle movement and pinching to assess elasticity, mobility, and adhesion to underlying tissue	1 = normal, 2 = supple (slightly reduced flexibility), 3 = yielding (moderate stiffness), 4 = firm (marked stiffness), 5 = contracture (immobile, adherent)
Surface area	Measurement using a ruler for the longest and perpendicular dimensions	10-point scale from 1 = minimal scar (almost no visible) to 10 = scar covering entire affected area

Postoperative CT scans were performed at six weeks using the same parameters as the preoperative scans to assess fracture reduction and implant stability. Two radiologists independently evaluated the scans, and inter-observer reliability was assessed using the ICC to ensure consistency in radiographic findings.

Statistical analysis

Descriptive statistics included means and standard deviations for continuous data, as well as frequencies for categorical data. The normality of continuous data was confirmed using the Shapiro-Wilk test. For longitudinal pain assessment, repeated-measures ANOVA was employed, with post hoc Bonferroni correction applied for inter-time point comparisons. Differences in the POSAS 3.0 scores on days 10 and 28 were evaluated using paired t-tests. The complication rate of the surgical procedure was analyzed using the chi-square test. Statistical significance was set at p < 0.05 for all tests. Data analysis was performed using SPSS Statistics version 27.0 (IBM Corp., Released 2020. IBM SPSS Statistics for Windows, Version 27.0. Armonk, NY: IBM Corp.).

## Results

The cohort consisted predominantly of males, with 12 (80%) male patients and three (20%) females. The age distribution was as follows: one (6.67%) aged 18-20 years, four (26.67%) aged 21-30 years, six (40%) aged 31-40 years, and four (26.67%) aged 41-50 years. The primary cause of injury was road traffic accidents, accounting for 12 (80%) cases, while interpersonal conflicts were responsible for 3 (20%) cases. Fracture types included right ZMC fractures in seven (46.67%) patients, left ZMC fractures in five (33.33%), ZMC with maxillary involvement in two (13.33%), and ZMC with mandibular involvement in one (6.67%). The incision times for the Y-modified transconjunctival approach were as follows: 20 seconds in three (20%) patients, 25 seconds in five (33.33%), 30 seconds in four (26.67%), and 35 seconds in three (20%). Postoperative complications were minimal, with only one case (6.67%) of enophthalmos reported; the remaining 14 patients (93.33%) experienced no complications. The Y-modified transconjunctival approach demonstrated high efficacy, characterized by a low complication rate (6.67%) and predominantly uneventful healing. The technique's efficiency, as reflected in the short incision times, supports its clinical utility. The predominance of road traffic accident-related injuries highlights the importance of targeted trauma-prevention strategies. Further research with a larger sample size is recommended to validate these findings (Table 2).

**Table 2:** Descriptive statistics of the study parameters. Data is presented in the form of n (%). ZMC: zygomaticomaxillary complex

Parameters	Category	n	%
Sex	Male	12	80.00%
	Female	3	20.00%
Age group (years)	18-20	1	6.67%
	21-30	4	26.67%
	31-40	6	40.00%
	41-50	4	26.67%
Type of injury	Interpersonal conflict	3	20.00%
	Road accident	12	80.00%
Fracture diagnosis	Right ZMC	7	46.67%
	Left ZMC	5	33.33%
	ZMC with maxilla	2	13.33%
	ZMC with mandible	1	6.67%
Time taken for incision (sec)	20	3	20.00%
	25	5	33.33%
	30	4	26.67%
	35	3	20.00%
Complications	Enophthalmos	1	6.67%
	No complications	14	93.33%

The comparison of pain scores, measured using the VAS, at different postoperative intervals for the 15 patients undergoing the Y modification of the transconjunctival approach for ZMC fractures is presented in Table 3. On POD 1, the mean VAS score was 5.82 ± 0.98, decreasing to 2.82 ± 0.98 on POD 10, 1.64 ± 0.67 on POD 28, and 1.00 ± 0.00 on POD 42. Statistical analysis using ANOVA revealed a significant overall difference in VAS scores across the intervals (p = 0.01), indicating a progressive reduction in pain over time. The significant decrease in the VAS scores from POD 1 to POD 42 (p = 0.01) demonstrated effective pain management and recovery following the Y-modified transconjunctival approach. The near-complete resolution of pain by POD 42 (mean 1.00 ± 0.00) suggested minimal residual discomfort, highlighting the procedure's tolerability. These findings support the efficacy of the technique in achieving favorable postoperative outcomes, although larger studies are needed to validate pain trends and assess the long-term effects.

**Table 3:** Comparison of pain scores using VAS at different intervals. *p < 0.05: significant using repeated ANOVA. Data is presented in the form of mean and SD. POD: postoperative day, SD: standard deviation, ANOVA: analysis of variance, VAS: Visual Analog Scale [15]

Days	Mean	SD	F-stats	p-value using repeated ANOVA
POD 1	5.82	0.98	115.66	0.001*
POD 10	2.82	0.98		
POD 28	1.64	0.67		
POD 42	1.00	0.00		

Bonferroni analysis confirmed a statistically significant reduction in pain from POD 1 to POD 42, except between POD 28 and POD 42, indicating that pain was stabilized by POD 28. These findings underscore the efficacy of Y modification in achieving progressive pain relief with minimal discomfort for 6 weeks, supporting its clinical utility. Larger studies are required to confirm these trends (Table 4).

**Table 4:** Pairwise comparison of pain scores using post hoc Bonferroni analysis. *p < 0.012: significant, p-value adjusted for multiple comparisons. POD: postoperative day, CI: confidence interval

Paired group	Mean difference	CI at 95%	t-stats	p-value
POD 1 vs. POD 10	-3.0000	-3.8797 to -2.1203	7.42	0.001*
POD 1 vs. POD 28	-4.1800	-5.0597 to -3.3003	12.32	0.001*
POD 1 vs. POD 42	-4.8200	-5.6997 to -3.9403	16.03	0.002*
POD 10 vs. POD 28	-1.1800	-2.0597 to -0.3003	3.44	0.005*
POD 10 vs. POD 42	-1.8200	-2.6997 to -0.9403	6.43	0.001*
POD 28 vs. POD 42	-0.6400	-1.5197 to 0.2397	2.18	0.172

The observer-based POSAS 3.0 evaluation demonstrated significant improvement in all scar-related parameters between POD 10 and 28. Notably, the vascularity and pigmentation scores decreased from 2.36 to 1.37 and from 2.09 to 1.27, respectively (p = 0.001), indicating a reduction in redness and discoloration over time. Similarly, parameters reflecting scar contour and flexibility, including thickness, relief, and pliability, also showed significant reductions (p < 0.01), suggesting a progressive normalization of tissue architecture. The scar surface area decreased from 2.09 to 1.18 (p = 0.001), indicating notable contraction and healing of the incision site. Collectively, these findings highlight the favorable clinical progression of wound healing and the effectiveness of the surgical approach in minimizing postoperative scarring (Table 5).

**Table 5:** Observer evaluation using POSAS 3.0 on POD 10th and 28th compared with paired t-test. *p < 0.012: significant, p-value adjusted for multiple comparisons. Data is presented in the form of mean and SD. POD: postoperative day, SD: standard deviation, POSAS: Patient and Observer Scar Assessment Scale [16]

Parameter	POD 10 (mean ± SD)	POD 28 (mean ± SD)	t-stats	p-value
Vascularity	2.36 ± 0.50	1.37 ± 0.45	5.09	0.001*
Pigmentation	2.09 ± 0.70	1.27 ± 0.47	3.36	0.001*
Thickness	1.73 ± 0.90	1.18 ± 0.60	2.67	0.013*
Relief	1.73 ± 0.39	1.18 ± 0.40	3.41	0.002*
Pliability	1.73 ± 0.05	1.00 ± 0.00	50.57	0.002*
Surface area	2.09 ± 0.54	1.18 ± 0.40	4.69	0.001*

Patient-reported outcomes also indicated marked improvements in scar perception and symptoms from POD 10 to POD 28. Pain and itching scores significantly decreased from 2.55 to 1.45 and 2.36 to 1.36, respectively (p = 0.001), reflecting enhanced patient comfort over time. Other aesthetic and tactile parameters, such as color, stiffness, thickness, and irregularity, also showed statistically significant reductions (p = 0.001), underscoring a favorable subjective experience of scar healing. The complete resolution of irregularity by POD 28 (mean score: 1.00) suggested that the patients perceived their scars as nearly indistinguishable from the surrounding tissue. These outcomes reinforced the positive healing trajectory and high patient satisfaction associated with the surgical technique (Table 6).

**Table 6:** Patient evaluation using POSAS 3.0 on POD 10th and 28th compared with paired t-test. *p < 0.012: significant, p-value adjusted for multiple comparisons. Data is presented in the form of mean and SD. POD: postoperative day, SD: standard deviation, POSAS: Patient and Observer Scar Assessment Scale [16]

Parameter	POD 10 (mean ± SD)	POD 28 (mean ± SD)	t-stats	p-value
Pain	2.55 ± 0.52	1.45 ± 0.52	5.19	0.001*
Itching	2.36 ± 0.76	1.36 ± 0.67	3.42	0.002*
Color	2.27 ± 0.79	1.45 ± 0.52	3.00	0.006*
Stiffness	1.91 ± 0.83	1.18 ± 0.60	2.46	0.021*
Thickness	2.18 ± 0.60	1.27 ± 0.47	4.13	0.001*
Irregularity	1.91 ± 0.30	1.00 ± 0.00	10.50	0.001*

Of the 15 patients, enophthalmos was observed in 2 cases (13.3%), while 13 cases (86.7%) had an uneventful recovery. A chi-square goodness-of-fit test against a hypothesized complication rate of 5 % %showed no statistically significant difference (χ² = 2.193, p = 0.139) (Table 7).

**Table 7:** Complication rate of surgical procedure analyzed using chi-square test. p > 0.05: nonsignificant. Data presented as N = number of patients and percentage (%).

Complications	N	%	Chi-stats	p-value
Enophthalmos	2	13.3%	2.19	0.139
Uneventful	13	86.7%		
Total	15	100.00%		

## Discussion

The Y-modified transconjunctival approach for managing ZMC fractures with significant orbital floor discontinuity represents a promising advancement in maxillofacial trauma care. The findings of this study highlight the potential to address a challenging subset of injuries, where achieving adequate exposure and optimal outcomes is critical. For the 15 patients in our cohort, each navigating the physical and emotional toll of facial trauma, the Y-modification offered a pathway to recovery marked by minimal complications, effective pain resolution, and aesthetically pleasing results. These outcomes resonate deeply, as they reflect not only clinical success but also the restoration of confidence and quality of life for individuals affected by such injuries.

Our results demonstrated that a combination of ZMC and orbital floor fractures occurred more frequently in males compared to females, which aligns with previous studies [3,17]. Furthermore, road traffic accidents were the major cause of such fractures. Yamsani et al. [1] similarly identified road traffic accidents as the principal contributor in 11 (64.7%) of the subjects, followed by alterations in three (17.6%) and falls from elevation in three (17.6%). It was also noted that right-sided fractures were more common than left-sided fractures. The majority of the population exhibits right-handedness, with a mere 10% demonstrating left-handedness. Individuals exhibit a propensity to protect their right side as reflexive responses associated with self-preservation. In road traffic accidents, particularly for motorcycle riders or drivers, the right side of the face is more vulnerable due to its positioning relative to the impact zone, especially in countries with right-hand driving. A research investigation conducted by Rajkumar et al. [11] indicated a significantly elevated occurrence of right ZMC fractures (seven instances) compared to left-sided fractures (three instances).

The Y-modified transconjunctival approach for managing ZMC fractures achieved high efficacy, with a low complication rate of 6.67% (only one case of enophthalmos) and predominantly uneventful healing in 93.33% of patients. This aligns with prior studies [12,13] that highlight the transconjunctival approach, which is associated with reduced scarring and improved access to the orbital floor compared with traditional methods. Santosh and Giraddi have reported the transconjunctival approach as the most aesthetically pleasing method for accessing the infraorbital rim, orbital floor, and medial wall of the orbit [9]. In contrast, research conducted by Oztel et al. demonstrated that the subtarsal approach yielded non-visible scar formation in 61-76.5% of subjects, while simultaneously offering a direct and easily accessible route to the orbital rim [17]. Ridgway et al. [18] conducted an investigation using a transconjunctival surgical approach. They documented instances of entropion in two subjects over a decade-long observation period, attributing these findings to insufficient exposure within the field that necessitated considerable retraction.

The Y-modification conferred upon the surgeon enhanced access to both the inferior and lateral orbital rims, thereby eliminating the need for an additional lateral eyebrow incision. The resultant rectangular aperture formed by retraction of the Y incision in the transconjunctival approach afforded extensive and unobstructed visibility at both fracture locations. This assertion aligns with the findings of Tahernia et al. [14], who implemented the Y modification technique and asserted that concurrent exposure to both the inferior orbital and lateral orbital rims represented the most efficacious strategy compared to alternative methods. Similar results have been reported in other studies [11,13].

However, our focus on ZMC fractures with substantial orbital floor involvement, a subgroup that is often underrepresented in the literature, sets this study apart. Earlier research, such as that by Wang and Dillon [19], emphasized general ZMC fracture management or standard transconjunctival techniques, with less attention paid to complex cases that require orbital floor reconstruction. In contrast, our targeted approach underscores the ability of Y modification to provide enhanced exposure, enabling precise fracture reduction and orbital reconstruction, as evidenced by the significant correction of enophthalmos (p < 0.05).

The progressive reduction in pain, from a mean VAS score of 5.82 on POD 1 to 1.00 by POD 42, is particularly encouraging. For patients, this translates into a tangible improvement in daily comfort, allowing them to resume normal activities with minimal discomfort. The statistical significance of this trend (p = 0.01) supports the tolerability of the procedure. This finding aligns with those of Martinez et al. [13], who noted favorable pain outcomes with modified transconjunctival approaches. The near-complete resolution of pain by 6 weeks suggests that the Y modification not only facilitates surgical precision but also promotes a smooth recovery trajectory, which is a critical factor for patients grappling with the physical and psychological aftermath of trauma. The significant reduction in pain from POD 1 to POD 10 could be due to a reduction in postoperative edema, as noted by Shoukath et al. [20]. Nonetheless, El-Anwar et al. observed the presence of periorbital edema in all subjects undergoing subciliary and transconjunctival techniques within the initial postoperative week, with the transconjunctival cohort (which included lateral canthotomy) exhibiting more pronounced edema [21]. Periorbital edema during the early postoperative phase is attributed to injury sustained by the lacrimal drainage apparatus as a result of incisional and dissection procedures conducted in the vicinity of the lateral canthus [20].

Aesthetic outcomes, assessed using POSAS 3.0, further highlight the human impact of this technique. The significant improvement in scar parameters such as vascularity, pigmentation, thickness, relief, and pliability (p < 0.01) reflects a healing process that minimizes visible reminders of surgery. This is profoundly meaningful for patients, many of whom were young adults injured in road traffic accidents (80% of the cohort). A less noticeable scar can alleviate self-consciousness and foster a sense of normalcy, as evidenced by the complete resolution of scar irregularities on POD 28 (mean score: 1.00). Patient-reported outcomes, including reduced pain and itching (p = 0.001), reinforced this finding, suggesting high satisfaction and a positive subjective experience. Other authors have also reported that the Y-shaped approach results in the most subtle and well-camouflaged scar formation [11,12].

The efficiency of the Y-modified approach, with incision times ranging from 20 to 35 minutes, underscores its practicality in the operating room. This brevity minimizes surgical trauma and provides good exposure, which is particularly beneficial in patients with complex ZMC fractures. The technique's ability to expose critical anatomical landmarks, such as the frontozygomatic suture, infraorbital rim, and orbital floor, facilitates precise fracture reduction and implant placement, as confirmed by postoperative CT scans showing stable fixation. The prolonged time required for the transconjunctival technique with Y modification can be ascribed to the extensive cutaneous Y incision, which requires additional time for supplementary tissue dissection. Furthermore, the transconjunctival technique requires careful reconstruction of the lateral canthal tendon to prevent any misalignment of the eyelid, which may result from canthal asymmetry [11,13]. For surgeons, the Y modification provides a reliable tool for navigating the complexities of ZMC fractures. At the same time, for patients, it translates to a lower risk of complications and a faster return to normalcy.

The Y-modified transconjunctival approach offers several clinical advantages for managing ZMC fractures with orbital floor discontinuities. Their ability to provide enhanced exposure facilitates precise fracture reduction and orbital reconstruction, making them particularly suitable for complex cases. The low complication rate (6.67%) and high patient satisfaction, as evidenced by the POSAS 3.0 scores, supported its adoption in maxillofacial surgery. Surgeons can confidently employ this technique to achieve functional outcomes (e.g., correction of enophthalmos and resolution of diplopia) and aesthetic goals (e.g., minimal scarring), thereby improving patient quality of life.

Despite its strengths, this study has several limitations. The sample size of 15 patients, although adequately powered to detect enophthalmos correction, limited the generalizability of the findings. Larger multicenter studies are required to validate these results across diverse populations. A follow-up period of six weeks, which is sufficient for assessing early outcomes, does not capture long-term complications or scar maturation, which may evolve over months. In addition, the study’s focus on a single institution may reflect specific surgical expertise or patient characteristics that are not universally applicable. Future research should address these gaps by utilizing larger cohorts, extending follow-up periods, and incorporating more diverse demographic representations. The present approach was not compared with other established techniques, such as the subciliary or transconjunctival approaches without Y modification, limiting our ability to assess its relative efficacy. Furthermore, orbital volume, a critical parameter for assessing orbital floor reconstruction, was not quantitatively evaluated in this study. Randomized controlled trials with long-term follow-up and comprehensive outcome measures, including orbital volume assessment, are needed to validate these findings and establish a Y-modified approach among the existing surgical options. Future research addressing these gaps will enhance the evidence for optimizing maxillofacial trauma care.

## Conclusions

This prospective cohort study demonstrated that the Y-modified transconjunctival approach is highly effective and reliable for managing ZMC fractures with significant orbital floor discontinuity. The approach achieved a low complication rate of 6.67%, resulting in significant improvements in functional outcomes, including correction of enophthalmos and progressive pain reduction, which underscored the technique's efficacy in restoring orbital function and patient comfort. Aesthetically, the approach yielded favorable outcomes, with notable reductions in scar vascularity, pigmentation, thickness, relief, and pliability, along with high patient satisfaction, reflected in improved patient-reported POSAS 3.0 scores. These findings highlight the Y-modified transconjunctival approach as a promising option for addressing this challenging subset of maxillofacial injuries, offering a balance between functional restoration, aesthetic preservation, and minimal morbidity.

## References

[1] Yamsani B, Gaddipati R, Vura N, Ramisetti S, Yamsani R (2016). Zygomaticomaxillary complex fractures: a review of 101 cases. J Maxillofac Oral Surg.

[2] Tripathi GM, Sharma D, Gaharwar A, Gupta R, Shukla D, Shukla V (2016). Analysis of prevalence and pattern of zygomatic complex fractures in north-eastern part of Madhya Pradesh, India. Int J Contemp Med Res.

[3] Bradley D, Leung B, Saxena S, Dungarwalla M, Chapireau D, Fan K (2019). Surgical management of zygomatic complex fractures in a major trauma centre. Plast Aesthet Res.

[4] Courtney DJ (1999). Upper buccal sulcus approach to management of fractures of the zygomatic complex: a retrospective study of 50 cases. Br J Oral Maxillofac Surg.

[5] Nordgaard JO (1976). Persistent sensory disturbances and diplopia following fractures of the zygoma. Arch Otolaryngol.

[6] Mishra BP, Harish A, Mathew AM, Pradhan A, Sneha S, Murty V, Makkad RS (2023). Management of zygomatic fractures using different surgical approaches. Bioinformation.

[7] Farber SJ, Nguyen DC, Skolnick GB, Woo AS, Patel KB (2016). Current management of zygomaticomaxillary complex fractures: a multidisciplinary survey and literature review. Craniomaxillofac Trauma Reconstr.

[8] Kambalimath DH, Kambalimath HV, M.V.V. S, V. A Kumar, T.G. R, Deepak RM (2023). Retrospective analysis of management of zygomatic complex fractures. The Traumaxilla.

[9] Santosh BS, Giraddi G (2011). Transconjunctival preseptal approach for orbital floor and infraorbital rim fracture. J Maxillofac Oral Surg.

[10] Subramanian B, Krishnamurthy S, Suresh Kumar P, Saravanan B, Padhmanabhan M (2009). Comparison of various approaches for exposure of infraorbital rim fractures of zygoma. J Maxillofac Oral Surg.

[11] Rajkumar K, Mukhopadhyay P, Sinha R, Bandyopadhyay TK (2016). ‘Y’ modification of the transconjunctival approach for management of zygomatic complex fractures: a prospective analysis. J Maxillofac Oral Surg.

[12] Melek LN, Noureldin MG (2023). Zygomaticomaxillary complex fractures: finding the least complicated surgical approach (a randomized clinical trial). BMC Oral Health.

[13] Martinez AY, Bradrick JP (2012). Y modification of the transconjunctival approach for management of zygomaticomaxillary complex fractures: a technical note. J Oral Maxillofac Surg.

[14] Tahernia A, Erdmann D, Follmar K, Mukundan S, Grimes J, Marcus JR (2009). Clinical implications of orbital volume change in the management of isolated and zygomaticomaxillary complex-associated orbital floor injuries. Plast Reconstr Surg.

[15] Downie WW, Leatham PA, Rhind VM, Wright V, Branco JA, Anderson JA (1978). Studies with pain rating scales. Ann Rheum Dis.

[16] Carrière ME, Mokkink LB, Tyack Z (2023). Development of the patient scale of the patient and observer scar assessment scale (POSAS) 3.0: a qualitative study. Qual Life Res.

[17] Oztel M, Goh R, Hsu E (2021). Subtarsal versus transconjunctival approach: a long-term follow-up of esthetic outcomes and complications. J Oral Maxillofac Surg.

[18] Ridgway EB, Chen C, Lee BT (2009). Acquired entropion associated with the transconjunctival incision for facial fracture management. J Craniofac Surg.

[19] Wang HD, Dillon J (2021). Contemporary management of zygomaticomaxillary complex fractures. Semin Plast Surg.

[20] Shoukath S, Taylor GI, Mendelson BC, Corlett RJ, Shayan R, Tourani SS, Ashton MW (2017). The lymphatic anatomy of the lower eyelid and conjunctiva and correlation with postoperative chemosis and edema. Plast Reconstr Surg.

[21] El-Anwar MW, Elsheikh E, Hussein AM, Tantawy AA, Abdelbaki YM (2017). Transconjunctival versus subciliary approach to the infraorbital margin for open reduction of zygomaticomaxillary complex fractures: a randomized feasibility study. Oral Maxillofac Surg.

